# Ruxolitinib sensitizes ovarian cancer to reduced dose Taxol, limits tumor growth and improves survival in immune competent mice

**DOI:** 10.18632/oncotarget.21541

**Published:** 2017-10-04

**Authors:** Patrick M. Reeves, Mojgan A. Abbaslou, Farah R.W. Kools, Kritchai Vutipongsatorn, Xiaoyun Tong, Christina Gavegano, Raymond F. Schinazi, Mark C. Poznansky

**Affiliations:** ^1^ Vaccine and Immunotherapy Center, Division of Infectious Diseases, Department of Medicine, Massachusetts General Hospital, Boston, MA 02129, USA; ^2^ Center for AIDS Research, Laboratory of Biochemical Pharmacology, Department of Pediatrics, Emory University School of Medicine, Atlanta, GA 30322, USA

**Keywords:** Ruxolitinib, ovarian, Taxol, combination, immunocompetent

## Abstract

**Background:**

Chemotherapy initially reduces the tumor burden in patients with ovarian cancer. However, tumors recur in over 70% of patients, creating the need for novel therapeutic approaches.

**Methods:**

We evaluated Ruxolitinib, an FDA-approved JAK 1/2 kinase inhibitor, as a potential adjunctive therapy for use with low-dose Taxol (Paclitaxel) by assessing the impact on *in vitro* proliferation and colony formation of ID8 cells or human TOV-112D ovarian cancer cells, as well as flow cytometric measurement of surface markers associated with cellular stress and stemness by ID8 cells. The syngeneic ID8 murine model of ovarian cancer was used to assess the impact of Ruxolitinib and Taxol, individually and in combination, on tumor initiation and growth, as well as capacity to extend survival.

**Results:**

Ruxolitinib (≤10 μM) sensitized both ID8 and TOV-112D cells to low concentrations of Taxol (≤5 nM), limiting cell proliferation and colony formation *in vitro*. Mechanistically, we demonstrated that Taxol induced expression of stress and stemness markers including GRP78 and CD133 was significantly reduced by addition of Ruxolitinib. Finally, we demonstrated that a single administration of a low-dose of Taxol (10 mg/Kg) together with daily Ruxolitinib (30 mg/Kg; which is equivalent to plasma concentrations of ∼ 0.01 μM steady-state) limited ID8 tumor growth *in vivo* and significantly extended median survival up to 53.5% (median 70 v 107.5 days) as compared to control mice.

**Conclusion:**

Together, these data support the use of Ruxolitinib in combination with low-dose Taxol as a therapeutic approach with the potential for improved efficacy and reduced side effects for patients with recurrent ovarian cancer.

## INTRODUCTION

Many patients with ovarian cancer are diagnosed late in disease progression (stage III and IV), resulting in a 5-year survival rate of less than 30% [1, 2]. As a result of this, ovarian cancer is the fifth most lethal cancer for women [2, 3]. Standard treatment for ovarian cancer frequently involves tumor reduction surgery together with chemotherapy, which can significantly reduce tumor burden and extend survival. However, the majority of patients who receive traditional surgery and chemotherapy relapse less than 18 months after initial diagnosis. The high morbidity and mortality in those with rapid relapse highlights the need for additional robust therapies to complement surgical removal of tumor masses [2, 3]. Numerous approaches using targeted therapies to limit tumor growth and neovascularization remain under evaluation including Bevacizumab, an anti-vascular endothelial growth factor (VEGF) antibody, and Erlotinib, a small molecule inhibitor of epidermal growth factor receptor (EGFR) [4–6]. Another approach seeks to enhance the sensitivity of cancer cells to chemotherapy through the use of small molecules targeting essential signal transducing molecules such as AKT and Aurora kinases [7, 8]. Here, using both the TOV-112D human cell line and the immunocompetent murine ID8 model, we assessed the impact on ovarian cancer of Ruxolitinib (Ruxo, Jakafi), an FDA-approved, orally bioavailable small molecule inhibitor of Janus Kinases 1/2 (JAK 1/2), delivered alone or in combination with low-dose Paclitaxel (Taxol) chemotherapy.

JAKs are cytosolic tyrosine kinases linked to signaling from cell membrane receptors including G-protein coupled receptors and cytokine receptors that activate signal transducer and activator of transcription (STAT) family transcription factors to alter gene expression [9]. Tumors can express stress response genes as a result of hypoxia, mechanical tension, and toxins (including chemotherapeutic agents) which through JAK/STAT signaling and expression of stress-response genes is also associated with stemcell-like properties or stemness [10]. Several STAT-dependent stress response genes including GRP78, CD133, SCA-1 (CD44), and CD117 are upregulated in ovarian cancer [11–14]. This increased expression of stress-response and stem cell markers is associated with drug resistance, tumor initiation and progression, and metastasis in human breast and ovarian cancer [12]. Ovarian cancer exploits a variety of resistance mechanisms including target protein or compensatory mutations, expression of drug pumps, induction of stress-response genes, adoption of stem cell characteristics, immune evasion through recruitment of regulatory T-cells (T-regs), and chronic interferon signaling [15, 16]. Importantly, previous reports indicate that Taxol can induce expression of STAT-dependent stress markers, while inhibition of the JAK/STAT pathway reduces the expression of stress markers and enhances sensitivity of cancer cells to chemotherapy, including hepatocellular and ovarian cancers [17–19]. The JAK 1/2 inhibitor Ruxo, initially approved to treat myelofibrosis, has shown potential utility in HIV, arthritis, and is currently under evaluation in combination with chemotherapy (e.g. Paclitaxel, Capecitabine) for the treatment of pancreatic cancer, breast cancer, and a recently announced trial for ovarian cancer [20–25]. These findings are likely due to the immunomodulatory properties of Ruxo, which confers potent, specific inhibition of IL-6, TNF-α, IL-1α/β, CRP, and other pro-inflammatory/immunoregulatory cytokines *in vivo*, which indirectly alter the systemic milieu for various pro-inflammatory indications, resulting in cessation of clinical manifestation of symptoms [26, 27]. It is possible that blockade of JAK-STAT signaling in the context of ovarian cancer, when applied at concentrations that allow for block of pro-cancer signaling while maintaining functional adaptive immunity, could result in additional benefit *versus* traditional mono-therapies alone.

Here, we show that treatment of murine and human ovarian cancer cells with a combination of Ruxo and low-dose Taxol limits ovarian cancer cell growth and colony formation *in vitro*. Taxol induced the expression of markers associated with cellular stress responses and stemness, GRP78, CD133, SCA-1, and CD117, and co-administration of Ruxo mitigated some of these effects. Additionally, using the C57BL/6 ID8 syngeneic murine model, we demonstrate that Ruxo augments the capacity of low-dose Taxol therapy to limit tumor growth and extend survival in immunocompetent animals. Together these data suggest that Ruxo in combination with low-dose Taxol chemotherapy may improve outcomes for patients with ovarian cancer, in part through its impact on mitigating cancer cell stress responses to the chemotherapeutic agent.

## RESULTS

### Ruxo in combination with low-dose Taxol *in vitro* limits ID8 and TOV-112D cell proliferation

To test the capacity of Ruxo to sensitize cells to Taxol, we assessed the impact of each compound individually on cell proliferation using ID8 murine and TOV-112D human ovarian cancer (Supplementary Figure 1). The selected concentrations of Ruxo, 5-10 μM, had limited or no impact on cell proliferation and represent concentrations at or above all C_max_ and steady-state plasma concentrations for this drug for all FDA approved doses in humans [26, 27]. The Taxol concentrations employed, 1-5 nM, limited proliferation 10-50%, allowing enhanced sensitivity by Ruxo to be observed. In patients, standard dosing of Taxol for ovarian and other cancers utilizes 135-250 mg/m^2^, and reduced doses from 60-80 mg/m^2^. Studies with lung and ovarian cancer patients determined that a 24-h i.v. infusion of Taxol 135 mg/m or 250 mg/m results in an average plasma steady state concentration of 320 nM and 850 nM respectively [28, 29]. Thus, Taxol concentrations used in this study are 64-850 fold less, and represent a low dose of Taxol.

**Figure 1:**
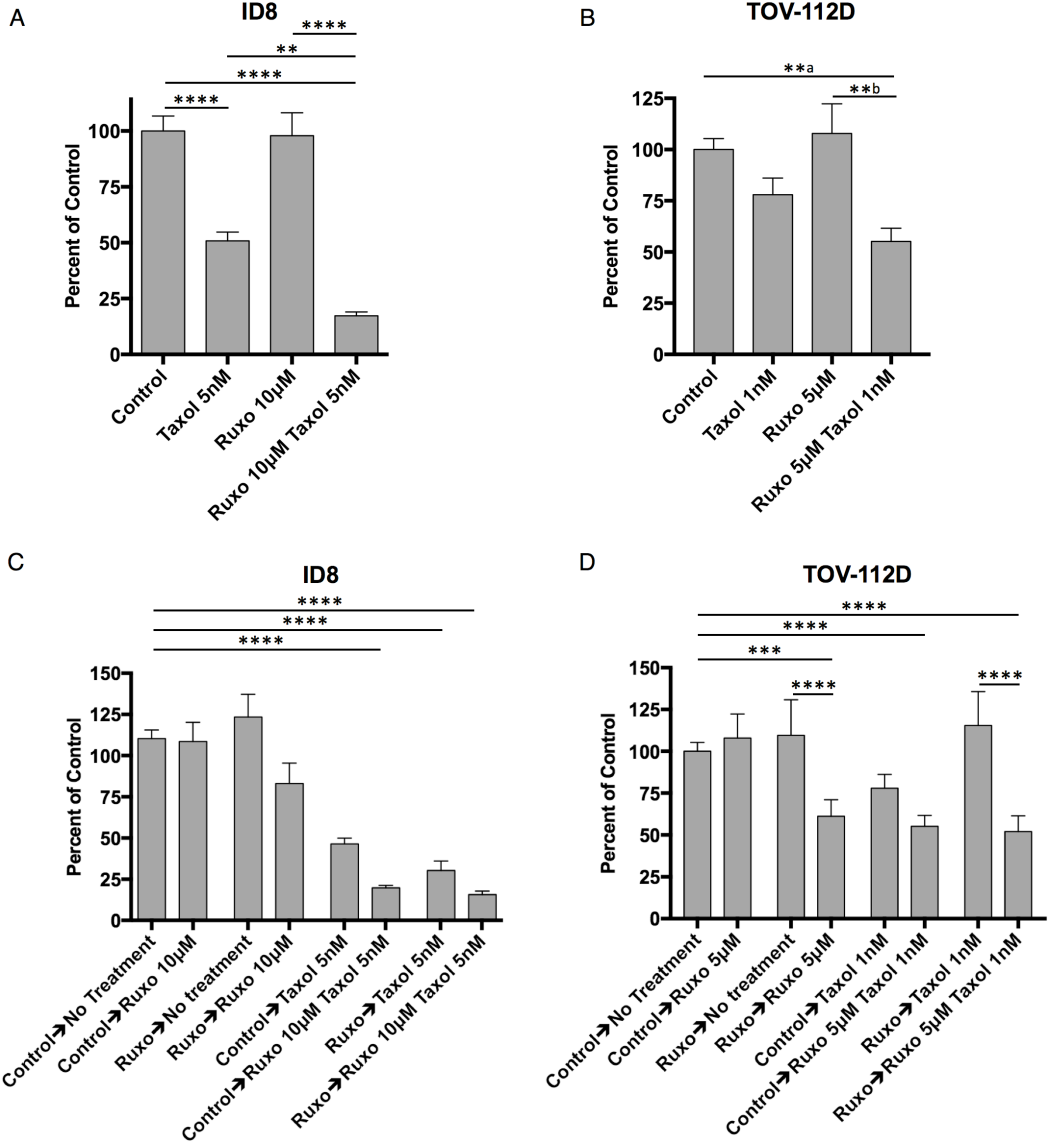
Ruxo and Taxol limit ID8 and TOV-112D cell proliferation **(A)** ID8 cells grown for 72 hours treated with carrier, 10μM Ruxo, 5 nM Taxol, or both, measured in triplicate (^**^ p = 0.0041, ^****^ p <0.0001, from 4 experiments) **(B)** TOV-112D cells grown for 96 hours treated with carrier, 5μM Ruxo, 1 nM Taxol, or both, measured in triplicate (^**^a p = 0.0097, ^**^b p = 0.0020, from 4 experiments). **(C)** ID8 cells were first exposed to carrier or Ruxo 10μM for 72 hours, harvested, and re-plated in triplicate in media containing carrier, 10μM Ruxo, 5 nM Taxol, or both for a further 72 hours. (^**^ p=0.0049, , ^****^ p <0.0001, from 3 experiments **(D)** TOV-112D cells were first exposed to carrier or Ruxo 5μM for 96 hours, harvested, and re-plated in triplicate in media containing carrier, 5μM Ruxo, 1 nM Taxol, or both for an additional 96 hours. (^***^ p=0.0003, ^****^ p <0.0001, from 3 experiments). Bars represent mean, error bars SEM. For panel A & B statistical analysis performed by one-way ANOVA followed by multiple comparisons between all groups with Bonferroni correction, in panels C & D two-way ANOVA followed by multiple comparisons between all groups with Bonferroni correction. Line above treatment groups indicates comparison resulting in statistically significant difference.

The proliferation of ID8 cells was not affected by Ruxo treatment alone even at concentrations as high as 10 μM. Taxol at concentrations as low as 5 nM proved sufficient to inhibit ID8 cell proliferation by 40-50% (Figure 1, Supplementary Figure 1). Co-incubation with both Ruxo (10 μM) and Taxol (5 nM) further reduced ID8 cell proliferation by as much as 90% (Figure 1A). In TOV-112D human ovarian cancer cells, Ruxo (5 μM) treatment alone did not significantly reduce cell growth, whereas low-doses of Taxol (1 nM) reduced proliferation by 22% (Figure 1B, Supplementary Figure 1). The combination of Ruxo (5μM) and Taxol (1 nM) reduced cell growth by 45% (Figure 1B). Thus, in both murine ID8 cells and human TOV-112D cells, Ruxo sensitized cells to treatment with Taxol *in vitro*.

Recent reports indicate that for combination drug therapy, the order in which drugs are administered can significantly alter efficacy [30–32]. To test this possibility with Ruxo and Taxol, ID8 or TOV-112D cells were initially incubated in the presence of either Ruxo or carrier (DMSO) for 96 hr, at which time equal numbers of viable cells were re-plated and treated with Ruxo, Taxol, or a combination of both drugs. As shown in Figure 1C, pretreatment of ID8 cells with carrier followed by Taxol at 5 nM reduced cell proliferation by 50%, whereas pre-treatment with Ruxo followed by Taxol reduced cell growth by 75%. In addition, pre-treatment with carrier or Ruxo followed by incubation with both Ruxo and Taxol reduced proliferation by 84% and 90%, respectively (Figure 1C). Pretreatment of TOV-112D cells with carrier followed by incubation with Taxol reduced proliferation by 22%, while Ruxo pretreatment prior to Taxol did not reduce cell growth. However, pretreatment with Ruxo followed by Ruxo, Taxol, or both reduced proliferation by 40%, 45%, and 48% respectively. Together these data indicate that pre-incubation with Ruxo does not improve the effects of subsequent incubation with Taxol or Ruxo and Taxol together.

### Ruxo together with low dose Taxol limits tumor cell colony formation *in vitro*

**Figure 2:**
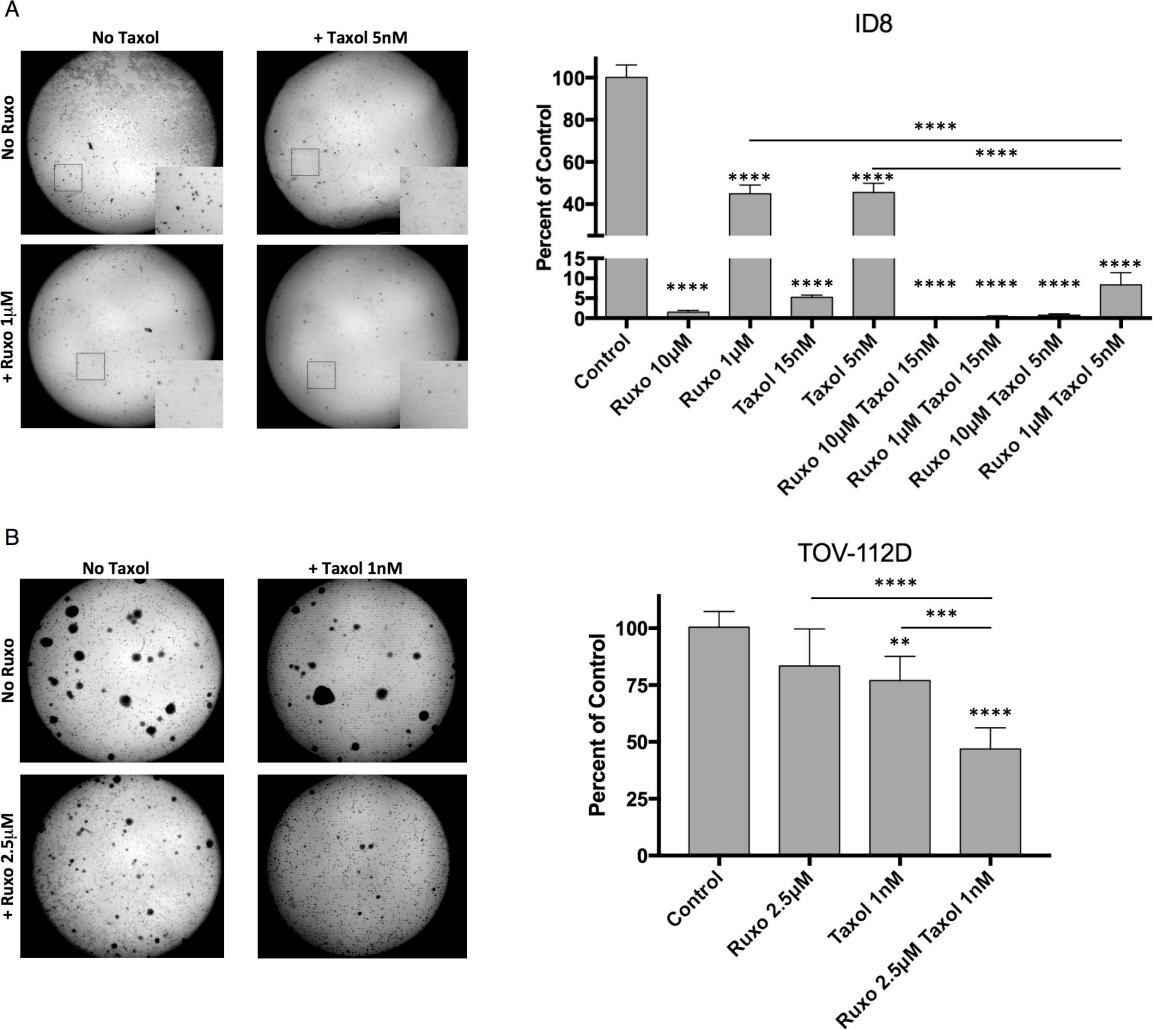
Ruxolitinib and Taxol limit ID8 and TOV-112D colony formation ID8 **(A)** or TOV-112D **(B)** cells were seeded in soft agar containing Ruxo or Taxol, or both. ID8 cells were treated with 10μM or 1μM Ruxo, 5 nM or 15 nM Taxol, or pairwise combinations of each. Regions matching 5x enlarged inset images indicated by white box. TOV-112D cells received 2.5μM Ruxo, 1 nM Taxol, or both. Plates were incubated for 30 days for ID8 and 21 days for TOV. Crystal violet stained colonies were imaged and subsequently enumerated on the basis of size using ImageJ, counting colonies ≥2mm for ID8 or ≥5mm for TOV-112D. Graphs represent normalized mean of 3 wells, error bar SEM, 3 independent experiments, one-way ANOVA analysis followed by multiple comparisons between all groups with Bonferroni correction. Significance relative to control indicated directly above condition. Inter-condition comparisons indicated by line (^**^ p= 0.0055, ^***^ p = 0.0006, ^****^ p <0.0001).

To further characterize the effect of treatment with Ruxo and Taxol on cell proliferation, soft-agar colony formation assays were carried out with ID8 and TOV-112D cells. Notably ID8 cells form smaller colonies (≥2mm) than TOV-112D cells which form larger colonies (≥5mm) and large clusters (≥15mm). Treatment of ID8 cells with Ruxo (10 or 1 μM) or Taxol (0.015 μM or 0.005 μM) alone reduced colony formation in a dose-dependent manner by 55-95% (Figure 2A). Co-incubation with both Ruxo and Taxol further reduced colony formation in a dose-dependent manner, demonstrating that co-incubation with both drugs is more effective than either drug alone (Figure 2A). In TOV-112D cells, Ruxo at 2.5 μM or Taxol at 1 nM alone reduced colony formation by 16% and 23% respectively, whereas co-incubation with Ruxo and Taxol reduced colony formation by 53% (Figure 2B). Together, these data demonstrate Ruxo and low-dose Taxol delivered in combination limit the capacity of mouse and human ovarian cancer cells to form colonies in soft agar.

### Ruxo limits Taxol induced expression of stress/stem cell markers in ID8 cells *in vitro*

**Figure 3:**
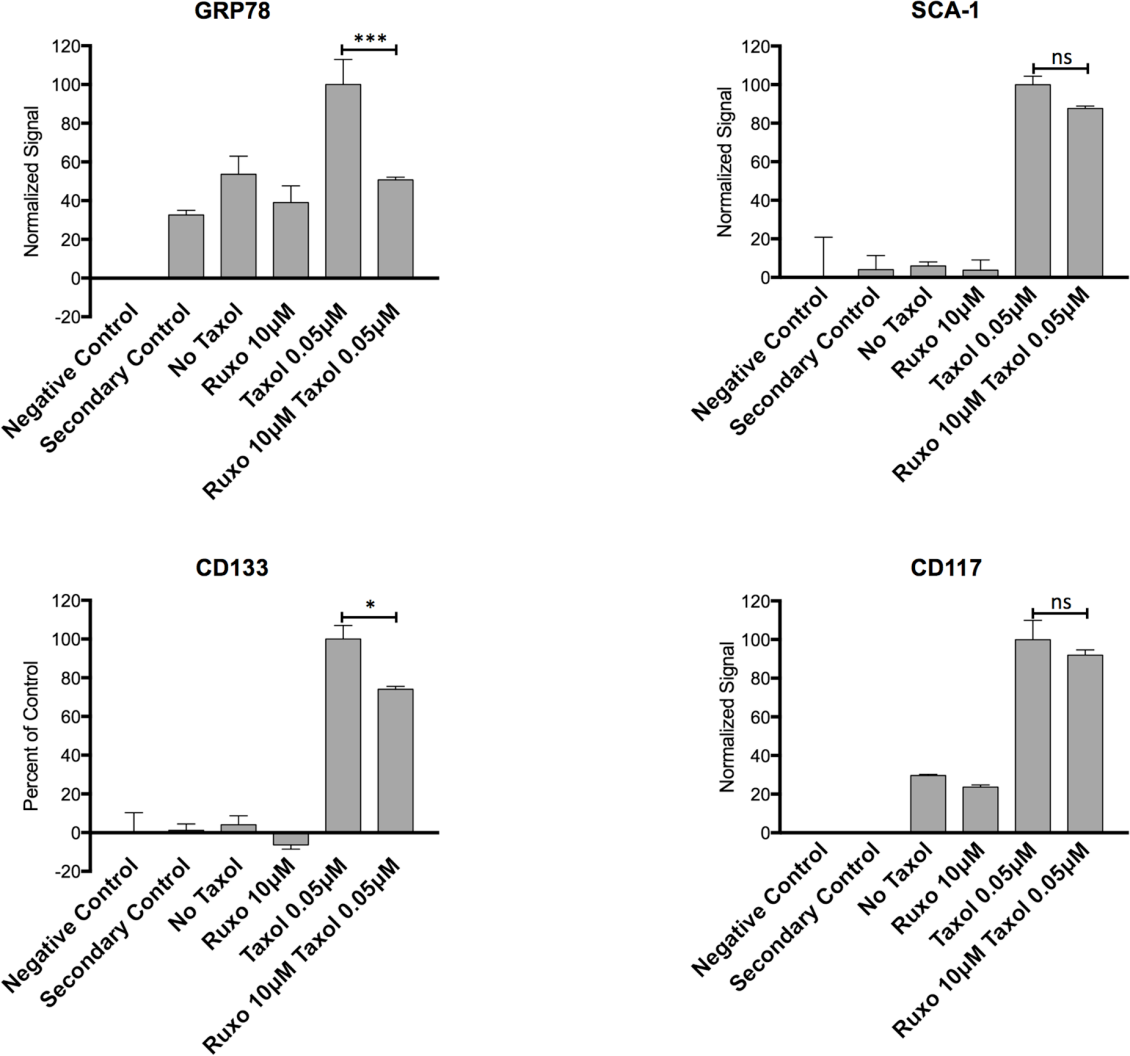
Effect of Ruxo and Taxol on Expression of stress and stemness associated markers ID8 cells were treated with Ruxo 10μM, Taxol 0.05μM or both for 96 hours and surface labeled with for GRP78 **(A)**, SCA-1 **(B)**, CD133 **(C)**, and CD117 **(D)**, negative controls are unlabeled and secondary control are cells labeled with secondary antibody only. Flow cytometry MFI values normalized by adjusting value of negative control (unstained cells) to 0 and Taxol 0.05μM to 100%. (^***^ p =0.0008, ^*^ p=0.0244). Graphs represent normalized mean of 2 wells, error bars represent SEM, representative of 2 independent experiments, one-way ANOVA analysis followed by multiple comparisons between all groups with Bonferroni correction.

To determine the effects of Ruxo and Taxol on the JAK/STAT induced stemness markers, surface expression levels of GRP78, CD133, SCA-1, and CD117, were assessed by flow cytometry. Changes in expression were measured in ID8 cells. Baseline levels, normalized to unlabeled cells, were assigned a value of 0%, whereas levels seen in cells treated with Taxol were assigned a value of 100%. Treatment with Taxol alone increased surface levels of GRP78, CD133, SCA-1, and CD117 relative to the untreated control by between 2 and 25 fold depending on the marker, whereas treatment with Ruxo produced at most modest reductions (0-15%) in surface levels of these markers (Figure 3). In contrast, treatment of ID8 cells with both Ruxo and Taxol significantly reduced the levels of GRP-78 (50%) and CD133 (30%) compared to Taxol treatment alone, levels of SCA-1 and CD117 were modestly reduced (<15%) (Figure 3). These data indicate that treatment with Taxol increases the surface expression of markers associated with the cellular stress response and stemness, and that co-administration of Ruxo can limit this effect.

### Ruxo and low-dose Taxol limit ID8 tumor growth *in vivo*

In view of the *in vitro* findings demonstrating that 1) Ruxo pretreatment confers potent block of ovarian cell proliferation, 2) addition of Ruxo and Taxol limit the capacity of ovarian cancer cells to form colonies, and 3) Ruxo mitigates the Taxol-induced up-regulation of pro-cancer markers (SCA-1, CD117), we sought to understand how these effects impact ovarian cancer *in vivo*. We assessed the impact of Ruxo and low-dose Taxol, delivered alone or in combination, on the capacity of ID8 cells to form tumors *in vivo* in immune competent mice*.* ID8 cells modified to express firefly luciferase (ID8-Luc) were first cultured *in vitro* with Ruxo 10 μM, Taxol 5 nM, both Ruxo and low-dose Taxol, or carrier (DMSO). Then, either 2.5x 10^5^ or 2.5x 10^6^ viable ID8-Luc cells from each pre-treatment group were injected intraperitoneally into C57BL/6 mice. Animals receiving post-tumor cell injection treatment were then administered Ruxo QD (30 mg/Kg) beginning 3 days post inoculation, whereas control (carrier only) animals were left untreated. Tumor size was monitored at weekly intervals for 5 weeks by *in vivo* imaging (IVIS) of luciferase signal. After this time, no further imaging was conducted and animals were monitored for survival.

**Figure 4:**
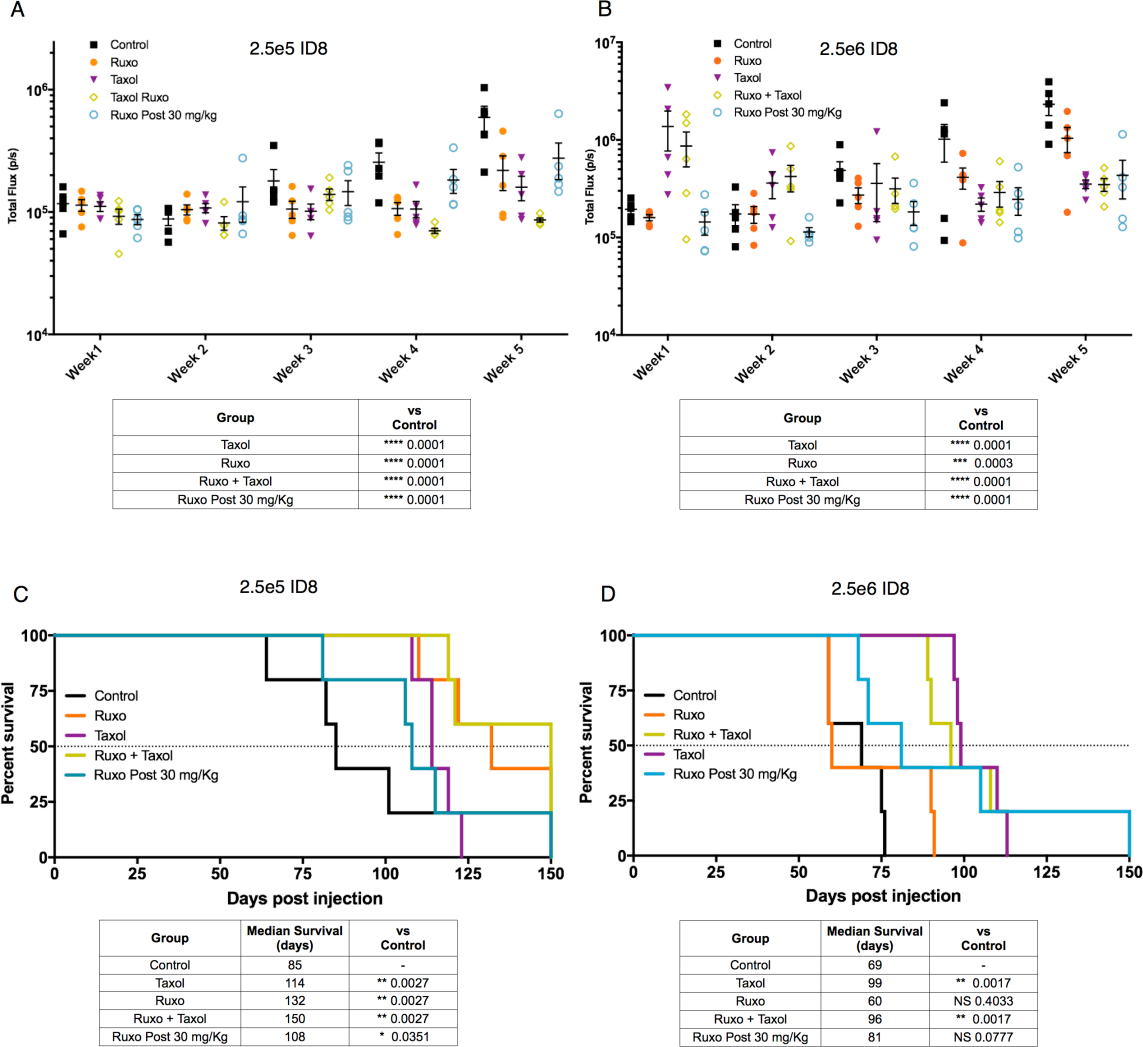
Effect of Ruxo and Taxol on ID8 tumor initiation and growth ID8-Luc cells treated with either carrier (control), Taxol, Ruxo, or Ruxo and Taxol in combination and then injected into mice either with 2.5x 10^6^ **(B** & **D)** or 2.5x 10^5^ **(A** & **C)** viable cells. In addition, one group received carrier treated control cells and daily Ruxo (30 mg/kg) days post injection. In panel (A & B) tumor growth was monitored weekly for 5 weeks as measured by luciferase imaging. Data points presented with median and SEM. Tables depict analysis of week 5 luciferase signal by two-way ANOVA followed by multiple comparisons between all groups with Bonferroni correction. In panels (C & D) survival data plotted, results of individual Mantel Cox test comparisons relative to control shown in table (N=5 per group, apart from 2.5x 10^5^ control N=4).

All cell pre-treatment conditions significantly (p ≤ 0.0003) reduced tumor burden at 5 weeks as compared to carrier-treated control cells (Figure 4A and 4B). In animals injected with 2.5x 10^5^ cells, tumors showed significantly less growth for all conditions relative to the control, with the Ruxo-Taxol group showing the greatest reduction (3.6 fold) in growth (Figure 4A). In animals receiving 2.5x 10^6^ cells, the Ruxo pre-treatment group showed less attenuation in growth (p = 0.0003), compared to the other groups, though all showed significantly less growth than the control group (all groups p <0.0001, Figure 4B)

In mice injected with 2.5x 10^5^ pre-treated cells, all treatments were associated with significantly increased survival relative to mice injected with control-treated cells (Figure 4C). Whereas the control group had a median survival of 85 days, the median survival for drug groups was between 1.2 and 1.8 fold longer (Ruxo = 132 days, Taxol = 114 days, Ruxo-Taxol = 150 days, and Ruxo post-treatment = 108 days) (Figure 4C). In mice injected with 2.5x 10^6^ cells, the control group had a median survival of 60 days and the median survival for the drug groups was 10 to 60% longer (Ruxo = 69 days, Taxol = 99 days, Ruxo-Taxol = 96 days, and Ruxo post-treatment = 81 days) (Figure 4D). The Ruxo-Taxol and Ruxo pre-treatment groups showed the greatest percentage increase in survival in both animals injected with 2.5x 10^5^ and 2.5x 10^6^ cells. In accordance with our tumor imaging studies, Taxol alone and Ruxo plus Taxol pre-treatment groups exhibited the greatest percentage increase in survival, achieving statistical significance in the 2.5x 10^6^ groups (p =0.0017 for both). These data indicate that Ruxo treatment alone, Ruxo in combination with Taxol, or administered daily post-inoculation, can limit tumor growth and prolong survival.

### Reduced doses of Ruxo and Taxol in combination extend survival in tumor-bearing mice

**Figure 5:**
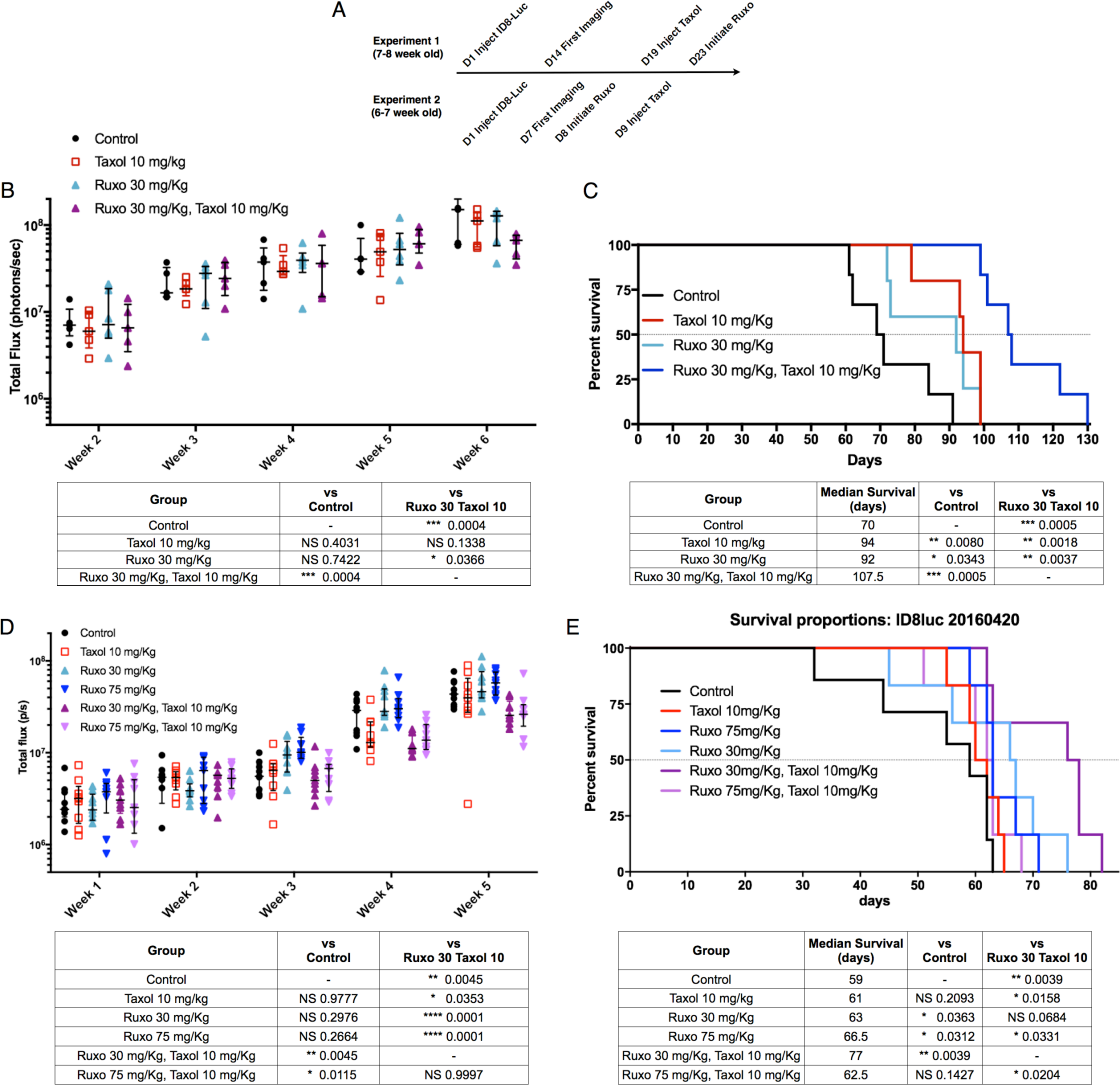
Impact of Ruxo and Taxol combination therapy on tumor growth and survival Mice were injected intraperitoneally with ID8-Luc cells **(A)** Timeline of studies described in panels B-D. Tumor growth was monitored weekly for 5 weeks as measured by luciferase imaging. For tumor growth in B & D, each data point is represented, with central line indicating median, upper and lower limits indicating quartile range. Results of two-way ANOVA followed by multiple comparisons between all groups with Bonferroni correction of final week values in table. Analysis of significance in survival studies determined using individual Mantel Cox test comparisons relative to control results are shown in table below graph (not significant = NS). **(B)** Experiment 1 tumor growth (N=5 Control, Taxol, Ruxo 30 mg/kg + Taxol, N=6 Ruxo 30 mg/kg). **(C)** Experiment 1 survival data and comparison analysis. (N=6 per group). **(D)** Experiment 2 tumor growth (N=8 control, N = 9 per treatment group). **(E)** Experiment 2 survival data and comparison analysis(N=6 per group).

To explore the therapeutic effect of Ruxo and Taxol in combination, the effects of reduced doses of either drug, alone and in combination, on tumor growth and survival were determined. We used a single administration of Taxol at a dose of 10 mg/Kg (Taxol 10), which represents approximately one third of the maximum tolerated dose in mice and is in the metronomic range [33, 34]. We followed two different treatment schedules, altering the duration of tumor growth prior to treatment, and the order in which Ruxo and Taxol were applied (Figure 5A). In the first experiment, mice (7-8 weeks old) were injected with 5x 10^6^ ID8-Luc cells. After 14 days, tumor growth was assessed weekly for 6 weeks by IVIS measurement of luciferase signal and treatment with a single dose of Taxol followed on day 19. Daily administration of Ruxo 30 mg/Kg/d (Ruxo 30), which corresponds to a human dose of ∼ 5-10 mg BID (pharmacokinetics performed as previously described [35]), began on day 23 post tumor inoculation. We observed that single agent Ruxo 30 or Taxol 10 treatment individually did not significantly reduce tumor growth by week 6. However, treatment with Taxol 10 followed by Ruxo 30 did significantly reduce tumor growth as compared to control (p = 0.0038) or as compared to single treatment with Ruxo 30 (p = 0.0366) but not Taxol 10 (p = 0.1338)(Figure 5B). Survival in mice treated with Ruxo 30 or Taxol 10 individually increased to 92 and 94 days respectively, versus 70 day median survival in the control group. Treatment with Ruxo 30 and Taxol 10 resulted in a median survival of 107.5 days, a 52.5% increase as compared to control (Figure 5C). Survival was significantly increased, with respect to control, for individual treatment with either Ruxo 30 (p = 0.0343) or Taxol (p = 0.008). The greatest increase in survival resulted from treatment with Taxol 10 followed by Ruxo 30 (p = 0.0005). Treatment with Taxol 10 and Ruxo 30 also significantly improved survival as compared to individual treatment with Ruxo 30 (p = 0.0018) or Taxol 10 (p = 0.0037).

In a second experimental approach, mice (6-7 weeks old) were injected with 5 x 10^6^ ID8-Luc cells. After one week, mice began treatment with either Ruxo 30/mg/Kg/d (Ruxo 30) or Ruxo 75 mg/Kg/d (Ruxo 75; which corresponds to 20-25 mg BID in humans; pharmacokinetics performed as previously described [35]). The following day, Taxol treated mice received a single administration of Taxol 10 mg/Kg. After 5 weeks, neither Ruxo 30, Ruxo 75, nor Taxol 10 treatment alone significantly inhibited tumor growth compared to control. In contrast, a single dose of Taxol 10 together with Ruxo 30 (p = 0.0045) or Ruxo 75 (p = 0.0115) significantly reduced tumor growth compared to control mice (Figure 5D). Treatment with Ruxo 30 and Taxol 10 significantly reduced tumor growth as compared to Ruxo 30 alone (p = 0.0001), similarly Ruxo 75 and Taxol 10 also significantly limited tumor growth as compared to Ruxo 75 alone (p = 0.0001). No difference in tumor growth was observed with Ruxo 30 and Taxol 10 as compared to Ruxo 75 and Taxol 10. The median survival was 59 days in the control group and treatment increased median survival to, 66.5 days for Ruxo 30 treated mice, 63 days for Ruxo 75 mice, and a single dose of Taxol 10 resulted in 61 days (Figure 5E). Combination treatment with a single dose of Taxol 10 followed by Ruxo 75 had a median survival of 62.5 days and was not significantly better than Ruxo 75 or Taxol 10 alone. Notably, the combination of Taxol 10 treatment followed by Ruxo 30 extended median survival to 77 days, a 30.5% improvement over control. Compared to Ruxo 30 alone, the increased survival in mice treated with a combination of Ruxo 30 and Taxol 10 was marginally significant (p = 0.0684), but was significant when compared to Taxol 10 treatment alone (p = 0.0158) and Ruxo 75 in combination with Taxol (p = 0.0204, Figure 5E). Together these data indicate that a reduced dose of Ruxo, that corresponds to a human dose of 5-10 mg BID and the lowest FDA-approved dose for this agent, potentiates the effect of reduced dose Taxol. In addition, these data support the view that the combination of Ruxo 30 and a single administration of Taxol 10 is associated with a significant benefit on both tumor burden reduction and survival.

## DISCUSSION

Here we demonstrate, for the first time, the use of Ruxolitinib (Ruxo), a FDA-approved JAK 1/2 inhibitor, to sensitize ovarian cancer to low-dose Taxol based chemotherapy both *in vitro* and *in vivo* in an immunocompetent orthotopic murine model. The use of small molecule inhibitors that target signaling pathways essential for cancer cell proliferation in conjunction with chemotherapy has garnered significant interest as a way to increase the efficacy of chemotherapeutic agents and limit development of resistance as compared to monotherapy. However, combination approaches can also increase the likelihood of drug toxicities and serious side effects. Previous reports have investigated Ruxo for the treatment of cancer in several animal models of head, neck, non-small cell lung carcinoma, and ovarian cancer [17, 36, 37] and describe the immune stimulating effect of reduced dose chemotherapy, including Taxol [38–41]. In this context, our preclinical data presented here in lends supports to the utility of the combination of both of these therapeutic agents in ovarian cancer.

*In vitro*, we sought to determine whether Ruxo treatment could sensitize cells to the effect of Taxol, rather than deliver an additive or synergistic therapeutic effect on cell growth. A supra-physiologic concentration of Ruxo (2.5 - 10 μM) was selected in order to explore this potential modulation of the therapeutic effect of Taxol [26, 27]. We found that Ruxo did not exhibit anti-proliferative activity in ID8 or TOV-112D cells cultured in plates at any concentration used. We found that Ruxo in combination with low-dose Taxol reduced proliferation of murine ID8 and human TOV-112D ovarian cancer cells and that Ruxo did indeed significantly increase the sensitivity of cancer cells to the anti-proliferative effect of Taxol. Previous studies of human ovarian cancer cells describe Taxol induced expression of markers associated with cellular stress and stem cell phenotypes and that this induction can be limited by the JAK inhibitor CYT387 [17, 42]. We hypothesized, from a mechanistic standpoint, that Ruxo inhibition of JAK 1/2 may similarly limit Taxol induction of expression of markers associated with stress and stemness. In agreement with previous studies, we observed that treatment with a low concentration of Taxol was sufficient to increase the expression of stress/stem markers GRP78, CD133, SCA-1, and CD117 in ID8 cells and that Ruxo significantly reduced the expression of GRP-78 and CD133, and modestly reduced SCA-1 and CD117 expression.

We then demonstrated that Ruxo limited the capacity of cells to form colonies in a soft agar assay, an *in vitro* measure thought to correlate strongly with tumorigenic potential [43]. Similar to our *in vitro* observations, cells pre-treated with Ruxo, Taxol, or the combination of the two agents, limited their capacity to initiate tumors *in vivo* in immune competent mice. The data from mice that received 2.5 x10^5^ cells demonstrate that pre-treatment with Ruxo or low-dose Taxol individually can reduce the capacity of cancer cells to form tumors, and that the combination of both treatments was most effective in this regard. Mice that received a larger number of cells (2.5 x10^6^) also demonstrated reduced tumor growth, though the benefit of pre-treatment with Taxol or the combination of Ruxo and Taxol was nearly identical, a difference that may result from the larger number of cells injected. Taken together, these data indicate that JAK 1/2 inhibitors, including Ruxo, can limit the expression of stress/stem markers by cancer cells induced by treatment with Taxol and thereby reduce tumorigenic potential suggesting that one mechanism of Ruxo sensitization to low-dose Taxol is mediated by the limitation of the capacity of cancer cells to adapt to stress.

Previous reports demonstrate that inhibition of JAK/STAT signaling reduces ovarian cancer growth *in vitro* and in an immunodeficient xenograft mouse model [17]. Here we explored the *in vivo* effects of Ruxo in an immune competent orthotopic syngeneic ovarian cancer model in light of the important role the immune system plays in the response to tumors. To understand the effect of low and high doses of Ruxo and their therapeutic effect *in vivo,* we evaluated Ruxo delivered either at a dose of 75 mg/Kg/d (Ruxo 75) or a reduced dose of 30 mg/Kg/d (Ruxo 30) to mitigate the potential for negative impact on anti-tumor immunity (equivalent to ∼ 5-10 mg bid Ruxo in humans). Monotherapy with Ruxo 30 or Ruxo 75 did not significantly limit tumor growth, though both treatments did confer a moderate survival benefit. Treatment with Ruxo 75 in combination with a single Taxol 10 administration did reduce tumor growth, though it did not provide a statistically significant survival benefit. Conversely, the combination of Ruxo 30 and a Taxol 10 treatment limited tumor growth, increased median survival, and improved survival significantly as compared to control mice, Taxol monotherapy, or Ruxo treatment alone. One possible explanation for the improved performance of Taxol 10 in combination with Ruxo 30 as compared to Ruxo 75 may result from the reported capacity of both Taxol and Ruxo to influence immune function. Administration of 30 mg/kg once-per-day in the murine system is analogous steady-state plasma drug concentrations to the lowest FDA-approved doses for this drug (5-10 mg BID, [35]) and may not interfere with adaptive anti-tumor immune function.

Taxol is reported to not only limit cancer cell division, but to also stimulate macrophages, TH1 T-cell responses, adjuvant immune responses to both tumor cell and defined antigens, and impair regulatory T-cell (Treg) function [38, 39, 44]. Moreover, low-dose administration of Taxol has been demonstrated to reduce T-reg and myeloid derived suppressor cell (MDSC) function, while enhancing both the number and interferon-ϒ (IFN-ϒ) production of CD4 and CD8 T-cells within tumors [40]. Conversely, administration of Ruxo 75 mg/Kg to mice (which is akin to the highest FDA-approved doses for this agent; 20-25 mg BID) can limit DC activation and stimulation of antigen specific T-cell responses [35, 45]. Recently, Benci et al. reported that Ruxo treatment can disrupt JAK/STAT mediated chronic intra-tumoral interferon signaling and restore sensitivity to checkpoint blockade therapy [46]. The authors note that optimization of Ruxo dose and regimen would be essential, particularly considering the important role of interferon signaling in anti-tumor responses [46]. Our study confirms the importance of reduced dose Ruxo in combination with low dose taxol and serves as a focus for future exploration of the effect of this combinatorial therapeutic approach on anti-tumor immunity.

Ruxo is under evaluation as a potential therapy for solid tumors in several clinical trials. These trials utilize Ruxo doses ranging from 10 mg daily to 25 mg BID. Our data suggest that studies to evaluate the efficacy of Ruxo treatment for solid tumors, particularly in combination with chemotherapy and potentially low-dose Taxol, may observe improved responses through reduced dosing of Ruxo as opposed to the maximum tolerated dose or doses used to treat myeloproliferative disorders. Our study provides preclinical data demonstrating that treatment of ovarian cancer with Ruxo can sensitize cancer cells to chemotherapy, limit their stress/stem response, reduce tumor initiation and growth, and extend survival of tumor-bearing mice. These data support further investigation into the application of this strategy to best benefit patients. Continued study of Ruxo in the context of syngeneic models will enable investigation of the impact of Ruxo and Taxol therapy on both tumor cell biology and immune cell function to inform the effective use of this drug combination in patients with ovarian cancer.

## MATERIALS AND METHODS

### Cell lines

ID8 cells, a clone of the MOSEC ovarian carcinoma (gift from Paul Terranova, Univ. of Kansas) and TOV-112D cells (ATCC CRL-11731), were cultured in DMEM supplemented with 10% FBS, 1% L-glutamine, and 1% Penicillin/Streptomycin at 37°C and 5% CO_2_. ID8-Luc cells were generated by transduction with MSCV-Hygro-Luc retrovirus and selected using 400 μg/mL Hygromycin for two weeks.

### Flow cytometry

ID8 cells were seeded in 6-well plates (500,000 cells/well), incubated for 96 hr, and harvested using enzyme free Dissociation Buffer (Thermo Fisher Scientific 13151014). Equal numbers of cells from each condition were labeled with antibody by incubation at 4°C on a rocker. After washing, where needed, cells were incubated with secondary antibody for 45 min, and washed twice prior to analysis. Labeled cells analyzed with a BD Fortessa and FlowJo software.

Antibodies: Anti-GRP78 (C-20) Santa Cruz Biotechnology. Anti-mouse Ly-6A/E (Sca-1) [D7], anti-CD133 [315-2C11], anti-CD117 (c-kit) [ACK2], anti-CD44 [IM7] from Biolegend, anti-goat alexa fluor conjugated antibody from Jackson ImmunoResearch.

### *In vitro* assays with Ruxo and Taxol

Paclitaxel (Taxol) was purchased from Sigma Aldrich. Ruxolitinib (Ruxo) obtained from Medchemexpress and LC Labs. For proliferation assays, cells were cultured in 96-well black assay plates with optically clear bottoms, including a standard curve plate. Plates were incubated at 37°C with 5% CO2, with all conditions in triplicate. In sequential delivery experiments (Figure 1C & 1D) of small molecules and chemotherapy ID8 or TOV-112D cells were exposed to Ruxo in a 6 well plate (2.5 x10^5^ cells/well) for 72 or 96 hr respectively. Cells were harvested, washed in fresh media, and cell viability determined with propidium iodide, and cell suspension diluted to contain the desired number of viable cells. For each condition 500 viable ID8 cells or 2500 viable TOV-112D cells were then seeded into a 96-well plate. Cells were then incubated in DMEM containing carrier, Ruxo, Taxol, or both for 72hr (ID8) or 96hr (TOV-112D). Proliferation was measured using CyQuant Kit (Invitrogen) and read on a Perkin Elmer Envision 2103. Drugs were not replenished during any of the experiments. Data was analyzed in Graphpad Prism 7.

### Colony formation assay

Agar stock was prepared in water for the top (0.6%) and bottom (0.8%) layers with SeaPlaque Agarose (Lonza, cat 50070). The agar and 2X DMEM was warmed in a 42°C water bath for 30 min prior to mixing in equal proportions in a 24-well plate, 0.5 mL of bottom agar was added, rocked to distribute agar, and allowed to solidify. During this time, ID8 cells were trypsinized in the hood and 4 x 10^4^ cells per mL suspension was made. Cell suspensions were then mixed with soft agar stock in the 42°C water bath (1:2). 0.5 mL of the cell/DMEM/agar mixture then was added to each well. Drug-DMEM stocks were made to achieve the proper concentrations: Ruxo (25 and 10 μM), and Taxol (0.015 and 0.005 μM). The total volume of each well was 1.1 mL. Plates were then incubated in a humidified incubator with 50% CO_2_ for 20-30 days. At the end of the experiment, each well was stained with 0.005% crystal violet in 15% ethanol (140 μL per well) and plates were incubated again for several hours. Micrographs taken using an Olympus CK2 microscope coupled to an Omax A3550U camera. Colonies equal or bigger than 2 mm (ID8) or 5mm (TOV-112D) were counted with the ITCN plugin for Image J.

### *In vivo* tumor generation, imaging, and treatment

Female C57BL/6 mice (6–8 week old) were obtained from Jackson Laboratories. All experiments were conducted in accordance with guidelines from the Public Health Service Policy on Humane Care of Laboratory Animals and were approved by the Institutional Animal Care and Use Committee of Massachusetts General Hospital under protocol 2008N000128. Mice were monitored daily for health and survival and were euthanized when recommended by staff veterinarians on the basis of signs of distress as determined by: hunched posture, decreased activity, increased respiratory rate, ruffled fur, and progressive ascites formation.

In all *in vivo* experiments, ID8-Luc cells were washed twice in phosphate-buffered saline (PBS), harvested with non-enzymatic disassociation buffer, collected by centrifugation at 400 *g* for 5 min. Cells were passed over a 70 μm filter, enumerated, and viability determined using propidium iodide, and cell concentration adjusted to inject 200 μL of PBS containing viable ID8-Luc intraperitoneally (IP) into female C57BL/6 mice.

Tumor-bearing mice were imaged once a week for 5 weeks with an IVIS imaging system. Mice were injected with 100 μL D-luciferin Potassium Salt (30 mg/L, Regis Technologies) and after a 10 min incubation mice were imaged for 180s to determine luciferase activity from ID8-Luc tumor cells. To quantify luciferase activity from tumors, an identical Region Of Interest (ROI) was created for each animal and the total flux/s (photons per second) was measured for each ROI.

### *In vivo* tumor initiation model

ID8-Luc cells were cultured *in vitro* in the presence of Ruxo 10 μM, Taxol 5 nM, both Ruxo 10 μM and Taxol 5 nM, or carrier (DMSO) for 48hrs, harvested, and viable cells enumerated to deliver 5 x10^5^ or 5 x10^6^ viable cells IP.

### Tumor growth and survival model

Sub-confluent ID8-Luc cells were harvested as above to deliver 5 x10^6^ viable ID8-Luc cells to each mouse. Seven days post-injection mice tumor burdens were determined by imaging and mice were equally distributed across treatment groups. Ruxolitinib phosphate, either with 30 mg/Kg (Ruxo-Lo) or 75 mg/Kg (Ruxo-Hi), was delivered by oral gavage once daily A single Taxol treatment was performed by intraperitoneal injection of 0.2 mg Taxol solution in 100 μL. Treatment schedules were as indicated in Figure 5.

### Statistical analysis

All statistical analysis was conducted using Graphpad Prism 7. As indicated in figure legends, one-way or two-way ANOVA analyses were conducted followed by multiple comparisons with Bonferroni correction. Survival data was analyzed for median survival as well as individual survival curve comparisons to control conducted using Mantel-Cox test.

## SUPPLEMENTARY MATERIALS FIGURE



## Abbreviations

BID: bis in die (twice per day)
DMSO: dimethyl sulfoxide
EGFR: epidermal growth factor receptor
JAK: Janus Kinase
QD: quaque die (once per day)
Ruxo: Ruxolitinib
STAT: signal transducer and activator of transcription
Taxol: Paclitaxel
T-reg: regulatory T-cell
VEGF: vascular endothelial growth factor
