# Correction: Ruxolitinib sensitizes ovarian cancer to reduced dose Taxol, limits tumor growth and improves survival in immune competent mice

**DOI:** 10.18632/oncotarget.25791

**Published:** 2018-07-13

**Authors:** Patrick M. Reeves, Mojgan A. Abbaslou, Farah R.W. Kools, Kritchai Vutipongsatorn, Xiaoyun Tong, Christina Gavegnano, Raymond F. Schinazi, Mark C. Poznansky

**Affiliations:** ^1^ Vaccine and Immunotherapy Center, Division of Infectious Diseases, Department of Medicine, Massachusetts General Hospital, Boston, MA 02129, USA; ^2^ Center for AIDS Research, Laboratory of Biochemical Pharmacology, Department of Pediatrics, Emory University School of Medicine, Atlanta, GA 30322, USA

**This article has been corrected:** The corrected author name is given below:

**Christina Gavegnano**

Original article: Oncotarget. 2017; 8:94040-94053. https://doi.org/10.18632/oncotarget.21541

