# Supplementary material for: Ruxolitinib sensitizes ovarian cancer to reduced dose Taxol, limits tumor growth and improves survival in immune competent mice
Source: Oncotarget. 2017 Oct 4;8(55):94040–53. doi: 10.18632/oncotarget.21541 (PMC5706854; doi:10.18632/oncotarget.21541)
Supplement: Supplementary file 1 [file oncotarget-08-94040-s001.pdf]

Ruxolitinib sensitizes ovarian cancer to reduced dose Taxol and limits tumor growth and improves survival in immune competent mice

SUPPLEMENTARY MATERIALS

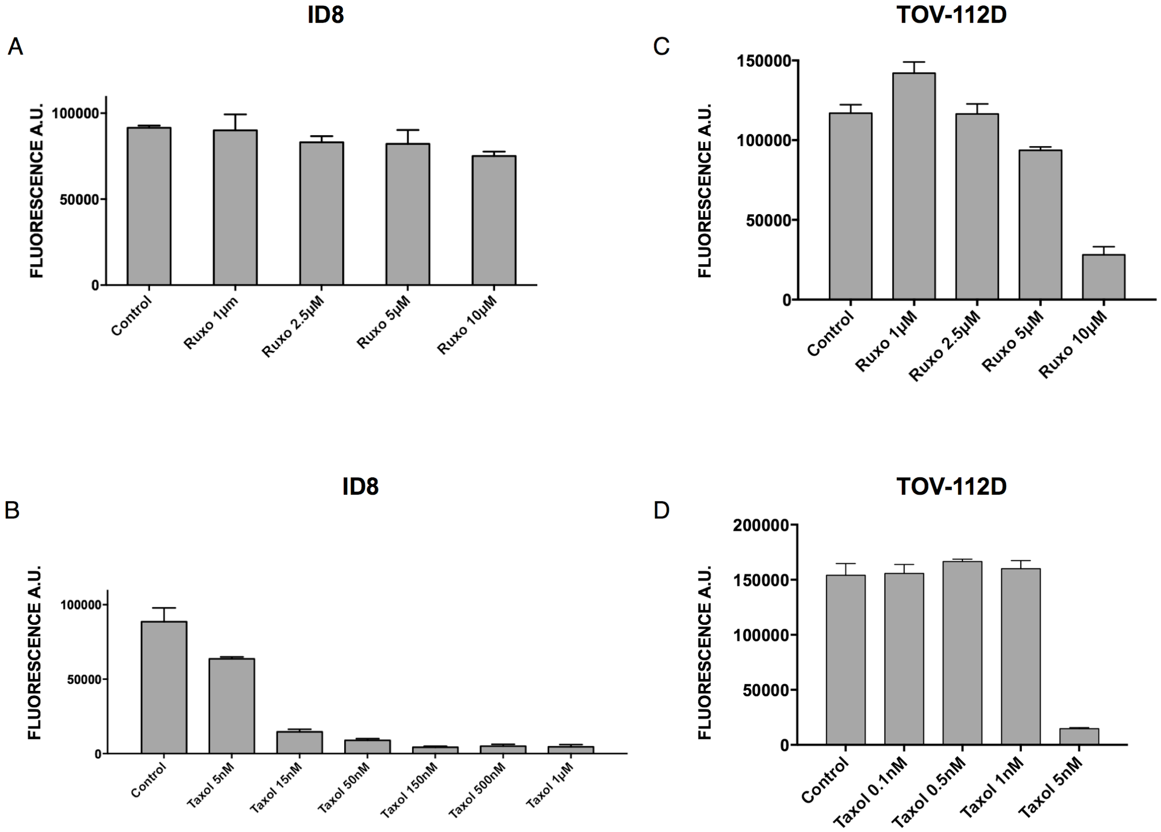

Supplementary Figure 1: Effect of Ruxo and Taxol on ID8 and TOV-112D cell proliferation. Cell proliferation was determined via CyQuant assay over a range of concentrations for both Ruxo and Taxol. **(A)** ID8 cells grown for 96 hours with DMSO control or 1-10 µM Ruxo measured in triplicate. **(B)** TOV-112D cells grown for 144 hours with DMSO control or 1 -10 µM Ruxo measured in triplicate. **(C)** ID8 cells incubated for 72 hours with carrier control or 1µM - 5nM Taxol measured in triplicate. **(D)** TOV-112D cells incubated for 144 hours with carrier control or 5nM - 1nM Taxol measured in triplicate. Bars represent mean, error bars SEM.
